# Benefits of applying hourly resolution in the assessment of the climate aptitude to manage tourist activities in arid regions

**DOI:** 10.1007/s00484-024-02685-7

**Published:** 2024-04-25

**Authors:** Fatemeh Nourmohammadi, María Belén Gómez-Martín

**Affiliations:** Department of Geography, University of Barcelona, Montalegre 6, Barcelona, 08001 Spain

**Keywords:** Nature-based tourism, Weather types method, Climate suitability, Tourism management, Iran

## Abstract

The availability of reliable information on local climatic-tourism conditions is a growing need due to the influence it exerts on the quality of the organizational strategy of tourist destination’s, and travel experience. Evaluations of the tourism potential of the climate have been carried out on a daily or monthly resolution, thus limiting the collection of detailed information that makes it possible to fine-tune tourism management and operational decision-making on an intraday scale. This research is the first case study to analyse the climatic suitability for nature tourism, using the weather types method at hourly resolution. The study applies to arid tourist destinations in Isfahan province (Iran). The detailed resolution has made it possible to identify the time slots favourable to the development of nature tourism in those periods of the year recognized as critical in the daily resolution analyses. In the same way, the hourly resolution has also identified critical bands in those periods indicated as favourable in the evaluations to daily resolution. The hourly resolution provides detailed information that can allow tourists and also tourism managers to establish intraday adaptation strategies that make it possible to develop the activity even in places with extreme climates.

## Introduction

Deserts are places of major natural, historical and cultural importance that have a special appeal for tourists. They offer an opportunity to travel and engage in leisure and recreational activities in wide-open, apparently boundless spaces featuring spectacular natural landscapes that are conducive to the enjoyment of a sense of freedom present in the motivations of most tourists (Narayanan and Macbeth 2009). In recent decades, tourism practiced in deserts has become a well-positioned niche attraction in the international market that responds to a wide range of tourism demand segments (Atkinson 2016). Indeed, this development has been examined in studies by Finzi et al. (2019) and Dinero (2002) for the Negev Desert in relation to activities linked to geological and cultural heritage, respectively; by Moufakkir and Selmi (2018) for the Sahara Desert in relation to the spiritual motivations of tourists; and by Różycki and Dryglas (2014) for the Namib Desert in relation to tourism and sporting practices such as trekking.

Grasping the potential and the defining characteristics of deserts is a key factor in bringing about their economic and social development. In this vein, the implementation of tourist activities in desert settings raises a number of critical issues that affect tourism production and consumption alike. The issues at stake include those related to the extreme climate, which poses risks to the safety and comfort of visitors, thereby affecting the design and programming of activities, tourism management, and destination planning (Faraj et al. 2023); those related to water scarcity, which hampers the management of water resources (Krakover 1985; Sulaiman et al. 2019); and those related to the limited population in deserts, which affects the involvement of local communities in tourism projects (Schmidt and Uriely 2019). Both the communication and transport difficulties and the geographical isolation can act as limiting factors in the development of mass tourism models (Kanwal et al. 2020), although, on the contrary, they may favour the development of locally based tourism that positions these characteristics as the main attraction (Laing and Crouch 2009). Thus, the climate seems to position itself as a determining factor that, on the one hand, defines biological, socioeconomic and cultural life in desert regions (Davis 2016; González Pizarro 2013) and, on the other, conditions -due to its character extreme- tourism management of the destination and the travel experience (Gómez-Martín 2005; Verbos et al. 2018).

### Arid climates and tourism

Not only tourists but also planners and operators of tourism destinations must adapt to the unfavourable climate conditions that deserts impose (Grigorieva 2021). Arid climates associated with desert ecosystems have characteristics that may ensure enjoyment but also, to a certain extent, put at risk the tourist’s requirements for comfort and safety (De Freitas 2003). The limited cloud cover, high daily hours of sunshine and lack of precipitation that define climates of this sort trigger physiological mechanisms that make the tourist feel joy, contentment and satisfaction (De Freitas 2015; Hoel et al. 2016). These aspects, which are a priori highly positive, can be jeopardised by extreme thermal conditions, low relative humidity in the environment, and a by no means negligible presence of wind that can imperil the comfort and safety of tourists. Tourists as human beings have a high adaptive capacity or resilience in the face of thermal stress that may result from the interaction of the indicated environmental conditions, the rate of physical activity (which determines the production of metabolic heat) and the clothing worn. Becoming acclimated to such extreme conditions involves not only significant physiological adjustments (Ciuha et al. 2019; Périard et al. 2015), but also behavioural adjustments, including a reduction or moderation of physical activity, the rescheduling of activities to other periods of the day, the use of climate shelters (interior or exterior spaces with benign microclimate conditions) and a change of apparel (Hewer 2020; Khalili et al. 2022; Périard et al. 2021).

To enable the adjustments in behaviour indicated above, it is crucial to furnish climate information aimed at the different actors involved in tourism. Meteorological and climate data are not sufficient on their own to lay a solid groundwork for decision-making: many studies have shown that the provision of suitable (adapted) tourism climate information has a direct influence on the quality of organisational strategies and operational decision-making (Damm et al. 2020; Gómez-Martín et al. 2017; Millán-López 2023). Against this backdrop, the particular characteristics of arid climates point clearly to the usefulness of preparing tourism climate timetables at different scales of space and time. From a spatial point of view, the climatic environment of the tourist centers depends on the local climate that derives from geographical factors that retouch in detail the regional atmospheric conditions (Barry and Blanken 2016; Besancenot 1991). Regional and local climate conditions are the ones that tourists consider when choosing a destination and organising their holidays. They are also the conditions that tourism planners and operators bear in mind when creating activity timetables and scheduling activities in destinations, forecasting the flows of visitors, managing potential climate emergencies and risks, launching marketing campaigns, setting the climate image of a destination, and calibrating the prices of tourism packages (Ayscue et al. 2015; Gómez-Martín 2005). In any case, it is important to note that the actual life of tourism plays out at the finer scale of micro-climates and local settings (Ali-Toudert et al. 2005; Besancenot 2013; Rutty and Scott 2014), which is an aspect that must be borne very much in mind in the design of strategies to adapt to the extreme and unpleasant atmospheric conditions of arid regions.

In terms of temporal resolution, the characteristics that typify arid climates warrant a twofold approach: daily and hourly. Applying daily resolution has been the most widespread and recommended approach in studies that evaluate the suitability of certain climates for tourism in order to determine which periods have the greatest tourism potential. Studies carried out for warm temperate areas of the Mediterranean (Gómez-Martín et al. 2002; Martínez-Ibarra 2008; Matzarakis et al. 2014; Nastos and Matzarakis 2019; Sahabi Abed and Matzarakis 2018) or for the humid tropics of the Caribbean or Asia (Gómez-Martín et al. 2020; Lin and Matzarakis 2011; Rutty et al. 2020; Zhao and Wang 2021), to name but a few examples, have typically used daily data grouped in periods of 30, 10 or 5 days according to the day-to-day variability recorded in the destinations, the characteristics of the method or index used in the evaluation, and the level of detail adopted in the research. The application of hourly resolution has been given less attention in tourism climatology (Matzarakis et al. 2013; Yu et al. 2009), although it is very important in the day-to-day scheduling of leisure and recreational activities in places with extreme climates. The extent of daily activity that can be pursued outdoors by tourists at a destination with an arid climate, however, takes into account neither the value of the average daily temperature nor the value of the daily high temperature, but rather the distribution of temperatures throughout the day. The same thing occurs with other variables such as the wind, precipitation and relative humidity (Dundas and von Haefen 2020; Fan et al. 2023). Unsurprisingly, it is common at tourism destinations in arid regions to find activities scheduled outside of what it is regarded as “the standard period of time for recreational activity”, which typically runs from 10 am until 6 pm. It is also necessary to add the fact that the experience of travel in the desert often involves spending the night outdoors at campsites.

### Evaluation of the suitability of arid climates for tourism

Evaluating the suitability of a climate for tourism can be conducted using indexes such as the Tourism Climate Index (Mieczkowski 1985), the Climate Tourism Information Scheme (Lin and Matzarakis 2008), the Holiday Climate Index (Yu et al. 2021; Díaz-Poso et al. 2023), the Climate Index for Tourism (de Freitas et al. 2008), the Beach Climate Index (Rutty et al. 2020) and the Beach Utility Index (Georgopoulou et al. 2019), to name but a few examples; or it can be done using the weather types method (Besancenot 1991; Gómez-Martín 2004, 2006; Gómez-Martín et al. 2020; Machete et al. 2014; Martínez-Ibarra 2008). The results arising from the application of different evaluation tools also depend on the procedures followed to establish the scales used in rating and weighting weather variables (whether for tourism in general or for different segments of tourism demand). Such procedures are sometimes grounded in expert judgement (Mieczkowski 1985) and sometimes based on bioclimatic criteria (De Freitas 1985; Freitas 1990). They may also reflect preferences expressed through surveys (Bafaluy et al. 2014; Denstadli et al. 2011; Gómez-Martín 2004, 2006; Hewer et al. 2018; Rutty and Scott 2013, 2015) and/or preferences revealed through behaviour (Gómez-Martín and Martínez-Ibarra 2012; Martínez-Ibarra 2011).

In the case of arid climates, applying the Tourism Climate Index (TCI) has been a popular approach in spite of the tool’s limitations (Scott et al. 2016; Rutty et al. 2020), which are related to: (a) the use of subjective criteria in the rating and weighting scales of the various sub-indexes that make up the TCI, without any empirical verification; (b) the failure to assess the dominance of physical variables in the index (for example, heavy precipitation and strong winds can cancel out the rest of the favourable weather conditions and yet such a possibility is not taken into account in the original version); (c) the failure to consider a variety of existing demand segments and their specific weather-related requirements, since the TCI is a generic index; and (d) the limited temporal resolution of the climate variables used in the tool (i.e. monthly averages). Using a monthly time scale is not good enough for tourism, although it is understandable since, as Scott et al. (2016) have pointed out, the TCI was developed at a time when standardised climate data was very hard to obtain. Recent studies continue making use of the TCI (Faraj et al. 2023; Farajzadeh and Matzarakis 2009; Masoudi 2021; Noome and Fitchett 2022), although some researchers now include modifications that consider the information on a daily basis (Hejazizadeh et al. 2019). The results of the latter studies, which are regional in coverage, show greater accuracy in the identification of months that are more suitable for tourism than the studies involving information on a monthly basis.

The Holiday Climate Index (HCI) has also been applied in regions with arid climates in order to identify the most suitable months for tourism (Hejazizadeh et al. 2019; Sobhani and Danehkar 2023). The HCI overcomes some of the limitations of the TCI, because it incorporates the climate preferences of tourists (collected by survey) in order to determine the rating scales and the relative weightings in the sub-indexes, and because it performs its calculations at the level of daily information. The HCI, however, does not take into account thermal comfort at night, since it takes the view that a large number of the establishments offering lodging have air-conditioning, an assumption that is not borne out on trips into deserts. The failure to consider this aspect gives rise to abnormal HCI values that are unrepresentative of the reality of desert settings, as research by Hejazizadeh et al. (2019) have demonstrated in the case of Iran.

The Outdoor Tourism Climate Index (OTCI) developed by Valizadeh and Khoorani (2020) also overcomes some of the TCI’s drawbacks, most notably by making it possible to work with monthly, daily and hourly data. Applying the tool to the dry, hot climate of Bandar Abbas (Iran), the authors provide evidence of the OTCI’s differentiated ratings as a function of the temporal resolution applied (in this case, monthly and daily resolutions). Applying daily resolution has a better fit with the reality of the settings in question than applying monthly resolution.

The Climate Tourism Information Scheme (CTIS) applied in Ghardaïa (Algeria, Sahara Desert) by Sahabi Abed and Matzarakis (2018) also provides evidence of the importance of high temporal resolution (daily) in the calculation of PET (Physiological Equivalent Temperature) and CTIS in settings of this sort. The grouping of the information in periods of ten days appears to be suitable for choosing the best period of year to travel, the destination, and the type of activities to pursue in such settings. The authors of the study point out that future studies conducted with hourly resolution could help to obtain more detailed information in order to schedule activities with the highest level of comfort, safety and enjoyment.

The weather types method has been applied recently in Iran in regions with arid and hyper-arid climates. In a study by Nourmohammadi and Gómez-Martín (2023), their consideration of the atmospheric reality of arid climates of Iran, with nuance added by considering bioclimatic criteria and the preferences of a specific segment of tourists, permits the definition of seven types of favourable weather for the practice of nature-based tourism (NBT). The day-to-day application of their proposed approach makes it possible to extract a value (in the form of a single figure that corresponds to the relative frequency of days that are likely to satisfy– to varying degrees– a tourist’s demands for enjoyment, safety and/or comfort) that encapsulates the suitability of a location’s climate, at any given time, for the pursuit of NBT. The approach helps to overcome the limitations that affect the most commonly used climate indexes, and it has the advantage of being based on the weather conditions actually experienced by the tourist. The information that results from the application of the method is very useful for operational management and decision-making by different tourism actors in the study area. As the study’s authors point out, however, an improvement in the temporal resolution could help to obtain even more detailed information that would be very beneficial for the organisation of activities in desert settings.

Both in the regional studies that have applied different tourism climate indexes using daily resolution and in studies that have applied the weather types method, the researchers identify some periods when extreme climate conditions are not suitable for tourism (periods coinciding with the summer months, due to high thermometric values and low comfort indices). Applying daily resolution and considering average values or values referring to the middle hours of the day might be masking favourable conditions that fall outside the midpoint of the standard period of recreational activity. Considering a higher temporal resolution might furnish very valuable information that could help to discern the time bands that are more favourable for leisure and recreation in these apparently unfavourable periods in desert settings.

The present study quantifies the suitability of the climate for nature-based tourism (NBT) in destinations with an arid climate in the Iranian province of Isfahan by applying hourly resolution with the weather types method. The analysis considers the preferences of domestic tourists in Iran. To date, there are no studies that have applied the weather type method on an hourly resolution, so this research is a novelty. The hourly timetables that are produced from the application of the weather types method offer a good tool for the provision of information both to tourists and the main tourism actors in the region, and can be very useful in destination management and decision-making.

## Study area

As Murgante et al. (2021) have indicated, desert tourism in Iran has a recent development in spite of the fact that more than two-thirds of the country’s total surface area is part of a desert ecosystem (Maghsoudi 2021). While the natural assets of these desert areas furnish a solid platform for the development of tourism in Iran, it has traditionally been the historical, religious and cultural heritage in urban areas that has accounted for tourism activity in the country. As Butler and Suntikul (2017) have pointed out, Iran is set up as a destination for religious, cultural and archaeological tourism for western tourists and for visitors from Islamic countries with similar beliefs. However, the development of medical tourism and nature-based tourism are attracting more and more interest among international customers and Iranians themselves.

In Iran, domestic tourism has received little attention in the literature and yet it exceeds international tourism in size (number of international tourist arrivals: 3,294,126 in 2011 and 8,832,050 in 2019; number of trips at domestic level: 54,797,940 in 2011 and 102,261,284 in 2019). Over the past three decades, the tourism sector in Iran has experienced a variety of problems that have affected the arrival of international tourists (Khodadadi 2016). Foremost among its problems are the country’s excessive economic dependence on petroleum and the limited diversification of the economy, the negative image of the country, international sanctions, political and social instability, and conflicts more broadly in the region of the Middle East where Iran is located. Seyfi and Hall (2019) show clearly that research into domestic tourism in Iran is fundamental for grasping the economic and cultural impacts that the activity generates at local and regional scale; for gauging its contribution to sustainable development in the region; and for evaluating the effectiveness of the policies implemented to that end. Doing research on the segment of Iranian domestic tourism interested in NBT in desert settings may be a key factor in developing attractive tourism products and engaging in suitable tourism planning and management in destinations that would benefit from development at local and regional scale.

**Fig. 1:**
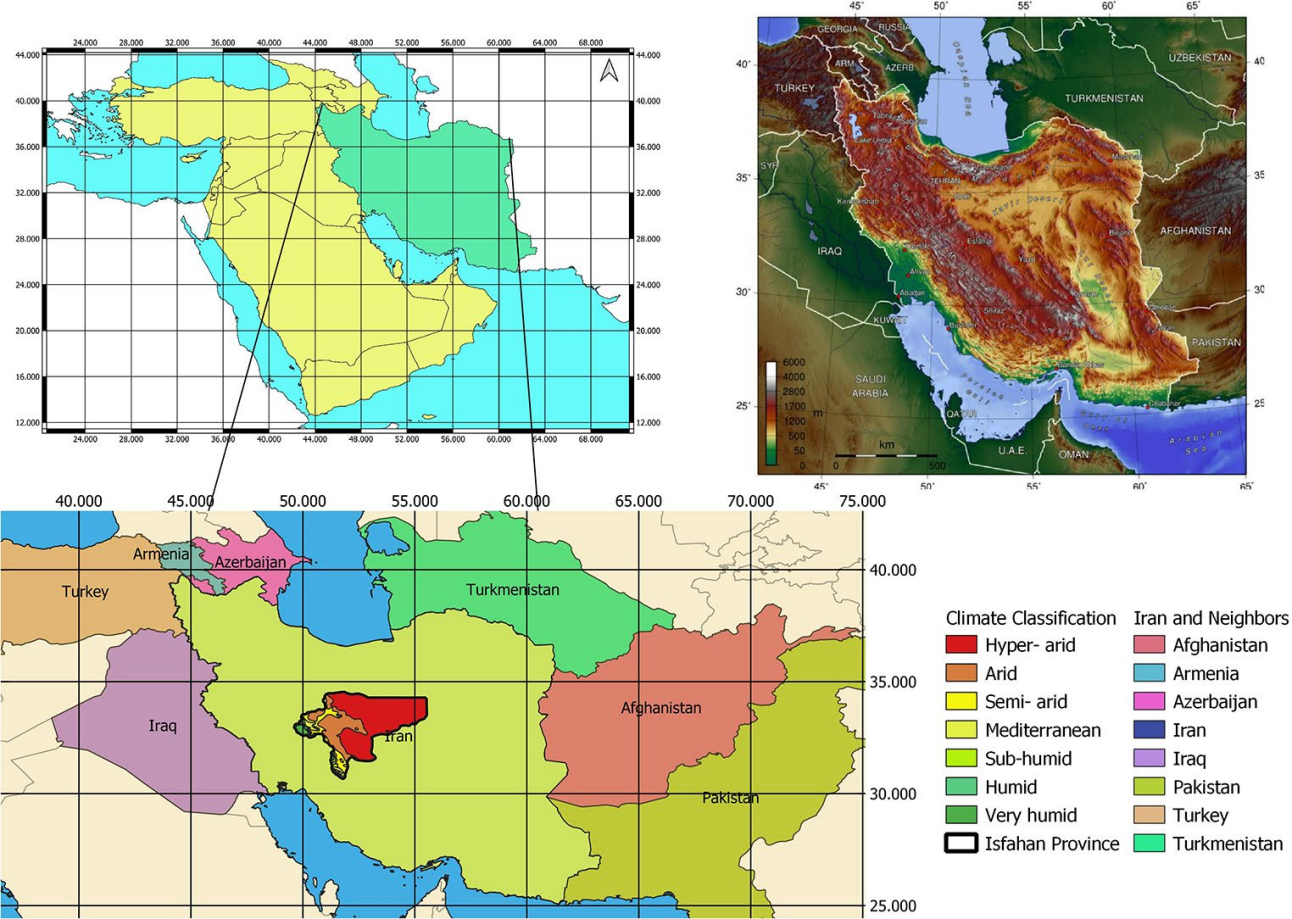
Location of the study area

The province of Isfahan, which is the study area for the present research, is an important tourist region in Iran (Fig. 1). The provincial capital, which is also called Isfahan, is a leading centre for cultural tourism, medical tourism and tourism related to conferences and incentives. The province also has a great potential for the development of nature-based tourism (NBT). More specifically, the province’s existing range of altitudes (which vary between 500 and 4,000 m) has given rise to a wide variety of landscapes and geographical phenomena, notably including the Varzaneh Desert, the Gavkhoni Wetland, the Maranjab Desert, Mount Soffeh, the valley of the Zayandeh River, the Neyasar Waterfall, the dunes of Rig-e Jenn and the Isfahan Desert. Current trends in tourism consumption give these places a position in the market and Iran has responded by creating nature-based products that include activities such as hiking, photography, stargazing, trekking through exceptional nature areas, off-road driving for 4 × 4 vehicles, and the observation of unique geological formations.

## Method and data

The method applied here starts by establishing a classification of weather types that are favourable for nature-based tourism in arid climates. The weather types have been defined on the basis of the characteristics of the climates in the area of study (IRIMO), together with bioclimatic criteria (Mahmoud 2011; Potchter et al. 2018) and the specific preferences of Iranian domestic tourists validated empirically by survey (Nourmohammadi Najafabadi and Gómez-Martín 2022).

The established weather types (see Table 1) take into account the tourists’ specific weather-related requirements in terms of enjoyment, comfort and safety. As a consequence, they build on the three facets of climate defined by De Freitas (2003) and Perry (1993), namely the aesthetic facet, evaluated on the basis of daily hours of sunshine; the thermal facet, evaluated on the basis of temperature and PET; and the physical facet, evaluated on the basis of precipitation amounts and wind speeds. The order of weather types in the ranking goes from most favourable (type 1) to least favourable (type 7), while type 8 lumps together all of the atmospheric conditions that are not suitable for nature-based tourism because they cannot guarantee the required levels of comfort, enjoyment and/or safety. In light of the specific characteristics of the climate in the area of study and the preferences of Iranian tourists, the approach here also establishes two distinct seasonal categories for weather types, specifically one for the months of summer (June, July and August) and another for the months of spring and autumn (respectively, March, April and May; and September, October and November). The results of our previous research (Nourmohammadi and Gómez-Martín, 2022) show that, with respect to nature-based tourism in regions with arid climates in Iran, domestic tourists have different climate preferences depending on the season of the year. The preferences in spring and autumn are similar but differ from the preferences declared for summer, mainly in the thermal values of the most favourable types of weather. This fact implies that climate potential assessments must seasonally adjust the rating scales for the different weather factors considered.

The applied method entails a modification of the approach put forward by Besancenot (1991), because it focuses on a highly specific segment of tourism demand and does not rely on expert judgement to define the thresholds for suitability among the atmospheric variables under scrutiny or the ranking of seasonal categories in play. As a result, the new classifications make it possible to evaluate the climate potential for nature-based tourism in the selected region while taking into account not only the climate preferences of Iranian tourists who travel in their own country but also the bioclimatic criteria involved. By doing so, the present adaptation overcomes the implicit limitations of the original method: it rectifies the absence of segmentation in the weather types method (the typology presented here is specific for the nature-based tourism segment) and the absence of empirical validation (both the ranking of weather types and the setting of thresholds for the variables refer to bioclimatic criteria and the preferences of Iranian domestic tourists interested in nature-based tourism).

After taking into account both the bioclimatic criteria and the preferences of Iranian tourists, the favourable temperatures have been set within the interval from 16 °C to 33 °C (where 16 °C corresponds to the point at which devices to combat cooling come into play and tourists at rest in light clothing begin to feel a chill, and 33 °C corresponds to the point at which the human body, with exposed skin, runs the risk of becoming unable to transmit sensible heat to the external medium) (Besancenot 1991; Escourrou 1980). More specifically, the proposed classification begins with type 1, which brings together the weather conditions regarded as optimal, namely a threshold of 20–23 °C in springtime and autumn and a threshold of 24–26 °C over the summer. For type 2, which covers weather conditions regarded as good, the thresholds are set at 24–26 °C in springtime and autumn and a threshold of 20–23 °C over the summer. For type 3, which covers good weather that is on the hot side, the thresholds are set at 23.1–29 °C for the spring, autumn and summer. For type 4, which covers good weather that is both hot and sultry, the thresholds are set at 29.1–33 °C for all seasons. For type 5, which covers good weather that is on the cool side, the thresholds are set at 14–19.9 °C for the spring, autumn and summer. For types 6 and 7, the thresholds are set at 14–33 °C because the drawbacks basically concern wind speed and rainfall.

Taking into consideration the length of the day or the maximum possible hours of sunshine in the area of study as well as the preferences of tourists (Nourmohammadi Najafabadi and Gómez-Martín 2022), the minimum hours of sunshine compatible with enjoyment stand at values greater than five hours a day not only during the summer but also in springtime and autumn. For type 8 (unfavourable weather), the thresholds fall below five hours of sunshine a day.

**Table 1:** Weather types for NBT in arid regions of Isfahan province

**Spring and autumn**					
	T	V_V_	*P*	S	PET
Type 1 Very good sunny weather	20 ≤ T ≤ 23 C°	V_V_ < 3.3 m/s	*P* = 0 mm	5 ≤ S ≤ 10 h	17.8 ≤ PET < 27 C°
Type 2 Fine weather with partial cloud cover	24 ≤ T ≤ 26 C°	V_V_ < 3.3 m/s	*P* = 0 mm	S ≥ 5 h	17.8 ≤ PET < 27 C°
Type 3 Fine hot weather	23.1 ≤ T ≤ 29 C°	V_V_ < 3.3 m/s	*P* = 0 mm	S ≥ 5 h	27 ≤ PET < 35.1 C°
Type 4 Fine hot and sultry weather	29.1 ≤ T ≤ 33 C°	V_V_ < 3.3 m/s	*P* = 0 mm	S ≥ 5 h	35.1 ≤ PET < 43 C°
Type 5 Fine cool weather	14 ≤ T ≤ 19.9 C°	V_V_ < 3.3 m/s	*P* = 0 mm	S ≥ 5 h	− 0.7 ≤ PET ≤ 17.7 C°
Type 6 Acceptable weather with strong winds	14 ≤ T ≤ 33 C°	3.3 ≤ V_V_ ≤ 5.4 m/s	*P* = 0 mm	S ≥ 5 h	-0.7 ≤ PET < 43 C°
Type 7 Acceptable weather with a brief rain shower	14 ≤ T ≤ 33 C°	V_V_ <3.3 m/s	0.01 ≤ *P* ≤ 5 mm	S ≥ 5 h	-0.7 ≤ PET < 43 C°
Type 8 Unfavourable / Bad weather	All other kinds of weather				
**Summer**					
Type 1 Very good Weather	24 ≤ T ≤ 26 C°	V_V_ < 3.3 m/s	*P* = 0 mm	S ≥ 5 h	17.8 ≤ PET < 27 C°
Type 2 Fine weather	20 ≤ T ≤ 23 C°	V_V_ < 3.3 m/s	*P* = 0 mm	5 ≤ S ≤ 10 h	17.8 ≤ PET < 27 C°
Type 3 Fine hot weather	23.1 ≤ T ≤ 29 C°	V_V_ < 3.3 m/s	*P* = 0 mm	S ≥ 5 h	27 ≤ PET < 35.1 C°
Type 4 Fine hot and sultry weather	29.1 ≤ T ≤ 33 C°	V_V_ < 3.3 m/s	*P* = 0 mm	S ≥ 5 h	35.1 ≤ PET < 43 C°
Type 5 Fine cool weather	14 ≤ T ≤ 19.9 C°	V_V_ < 3.3 m/s	*P* = 0 mm	S ≥ 5 h	− 0.7 ≤ PET ≤ 17.7 C°
Type 6 Acceptable weather with strong winds	14 ≤ T ≤ 33 C°	3.3 ≤ V_V_ ≤ 5.4 m/s	*P* = 0 mm	S ≥ 5 h	-0.7 ≤ PET < 43 C°
Type 7 Acceptable weather with a brief rain shower	14 ≤ T ≤ 33 C°	V_V_ <3.3 m/s	0.01 ≤ *P* ≤ 5 mm	S ≥ 5 h	-0.7 ≤ PET < 43 C°
Type 8 Unfavourable / Bad weather	All other kinds of weather				

In addition, rainfall has a well-known effect on the enjoyment and safety of tourism activities, especially outdoors in the open air. Precipitation levels, when they exceed certain thresholds, can act as a limiting factor that is able to eclipse any other favourable elements and all by itself give an impression of bad weather. Tourists prefer dry days, although they can go so far as to find days acceptable with 5 mm of rainfall or less (type 7). When rainfall is greater than 5 mm, however, a weather type is regarded as unfavourable (Nourmohammadi Najafabadi and Gómez-Martín 2022).

With respect to wind speed, the weather types that lend themselves favourably to nature-based tourism (types 1, 2, 3, 4, 5 and 7) can have velocities that do not exceed 3.3 m/s. Type 6 (good weather with strong winds), given its windy character, can have higher velocities that fall between 3.3 and 5.4 m/s, whereas the unfavourable type (type 8) covers values in excess of 5.4 m/s (Nourmohammadi Najafabadi and Gómez-Martín 2022).

Lastly, the scale used in PET readings takes into account an adaptation put into place for Iran by Farajzadeh (2017) and Sharafkhani et al. (2018). The PET calculation is made from the daily data, for every 3 h, of average temperature, wind speed, cloudiness and relative humidity. The personal data were: male, 35 years old, with a height of 1.75 m and a weight of 75 kg. In addition, clothing with a thermal resistance of 0.9 Clo and an activity of 80 W was considered. Accordingly, the values of maximum comfort for types 1 and 2 stand at 17.8–27 °C (comfortable). The thresholds for type 3, which covers good weather that is on the hot side, stand at 27–35.1 °C (slightly warm) for the spring, autumn and summer. For type 4, which includes good weather that is both hot and sultry, the thresholds are set at 35.1–43 °C for all seasons (warm). For type 5, which involves good weather on the cool side, the thresholds are set at between − 0.7 °C and 17.7 °C (slightly cool and cool). For types 6 and 7, the thresholds are set at between − 0.7 °C and 43 °C.

The information taken as a reference draws on the synoptic observations of the surface at the principal set times and intermediate set times (0000, 0600, 1200 and 1800 local time; and 0300, 0900, 1500 and 2100 local time, respectively) for the following variables at each of the times indicated: the temperature, in degrees Celsius; the accumulated precipitation, in mm; the relative humidity of the air, expressed as a percentage; and PET (ºC), calculated at each indicated time using the RayMan model proposed by Matzarakis et al. (2010). Regarding the wind speed, the average of the 10 min prior to the indicated time was calculated, in m/s. In the application of the catalogue, the figure for the amount of daily sunshine (in hours) is kept constant for all time bands.

The weather types method has been applied on an hourly basis for each year in the period 1998–2017. The weather stations of the Islamic Republic of Iran Meteorological Organization (IRIMO) that appear in the study are listed below in Table 2. It is important to note that the synoptic observatories at Naein and Natanz have not provided hourly information for 2100, 0000, 0300 and 0600 local time. The chronological variations in the atmospheric environment are shown in diagrams in which the recorded frequencies of favourable weather types (types 1, 2, 3, 4, 5, 6 and 7) are grouped at the indicated hours over periods of five days in length.

**Table 2:** Synoptic observatories included in the study

Synoptic observatories		J	F	M	A	M	J	Jl	A	S	O	N	D
Isfahan 32º 51’ 67’’ N 51º 70’ 56’’ E 1551.9 m	T T_MAX_ T_MIN_ P	3 9.4 -2.5 18.7	6 12.7 -0.1 15.3	10.7 17.3 4.3 22.3	16.1 22.8 9.4 20.3	21.4 28.4 14 8.8	27.1 34.5 18.7 1.3	29.5 36.9 21.2 1.7	27.9 35.7 19.2 0.4	23.7 31.9 14.8 0.1	17.1 25.1 9.1 4	9.7 16.9 3.2 15	4.4 11 -1 19.3
Kashan 33º 96’ 69’’ N 51º 48’ 08’’ E 955 m	T T_MAX_ T_MIN_ P	4.8 10.2 -0.7 25.1	7.8 13.6 1.4 18.3	13.3 19.4 6.4 25.8	19.9 26.4 12.1 17.4	25.5 32 17 12.4	31.3 38.1 22 1.5	34 40.8 24.9 0.4	32.5 39.8 23.3 0.5	27.7 35.3 18.5 0.2	20.5 27.6 12.6 4	12.6 18.7 6.1 14.1	6.7 12.1 1.1 16.8
Isfahan Airport 32º 74’ 41’’ N 51º 86’ 30’’ E 1550 m	T T_MAX_ T_MIN_ P	1.5 9.3 -5.6 15.5	4.5 12.8 -3.5 11.9	9.4 17.5 1.2 21.2	15.3 23.5 6.6 14.7	20.7 29.2 11 6.6	26.5 35.3 15.5 1	29 37.6 18.1 1.4	27.3 36.5 16.1 0.5	22.5 32.6 11.3 0.3	15.8 25.6 6.2 2.9	8.4 17.3 0.4 12.6	3.3 11.3 -3.7 16.9
Naein 32º 85’ 16’’ N 53º 07’ 86’’ E 1573.7 m	T T_MAX_ T_MIN_ P	4.9 9.6 -1.8 18.8	8 12.8 0.5 11.5	12.6 17.3 4.7 20.8	18.7 23.4 10.2 12.8	23.8 28.7 14.7 10.2	29.3 34.1 19.2 0.9	31.6 36.4 21.9 0.4	30.2 35.1 20 0.1	26.3 31.4 16 0.1	20 24.9 10.7 1.8	12 16.7 4.3 11.9	6.9 11.6 -0.1 11.6
Natanz 33º 53’ 33’’ N 51º 9’ 00’’ E 1685 m	T T_MAX_ T_MIN_ P	2.7 6 -1.9 33.4	5.4 9 0.2 22.8	10.3 14 4.6 38.6	16.4 20.1 10.3 28.8	21.7 25.5 15 17	27.4 31.2 20.3 2.6	29.9 33.8 23.3 0.5	29.1 32.9 22.4 0.6	24.8 28.6 18.4 0.4	18.3 21.8 12.5 4.9	10.1 13.3 5.3 22.4	4.9 8 0.6 21.5
Shahreza 31º 98’ 16’’ N 51º 81’ 05’’ E 1858 m	T T_MAX_ T_MIN_ P	2.8 8.8 -3.6 23.4	5.6 11.8 -1.1 18.7	9.9 16.1 2.5 26.1	15.3 21.4 7.5 18.2	20.6 27 11.5 7.1	26 32.8 15.4 1.8	28.6 35.1 18.5 0.6	26.9 33.9 16.1 0.3	22.5 30.2 11.6 0.9	16.3 23.8 6.5 3.9	9 15.7 1.5 21.4	4.3 10.8 -2.3 23

*Source* Iran Meteorological Organization

## Results

**Fig. 2:**
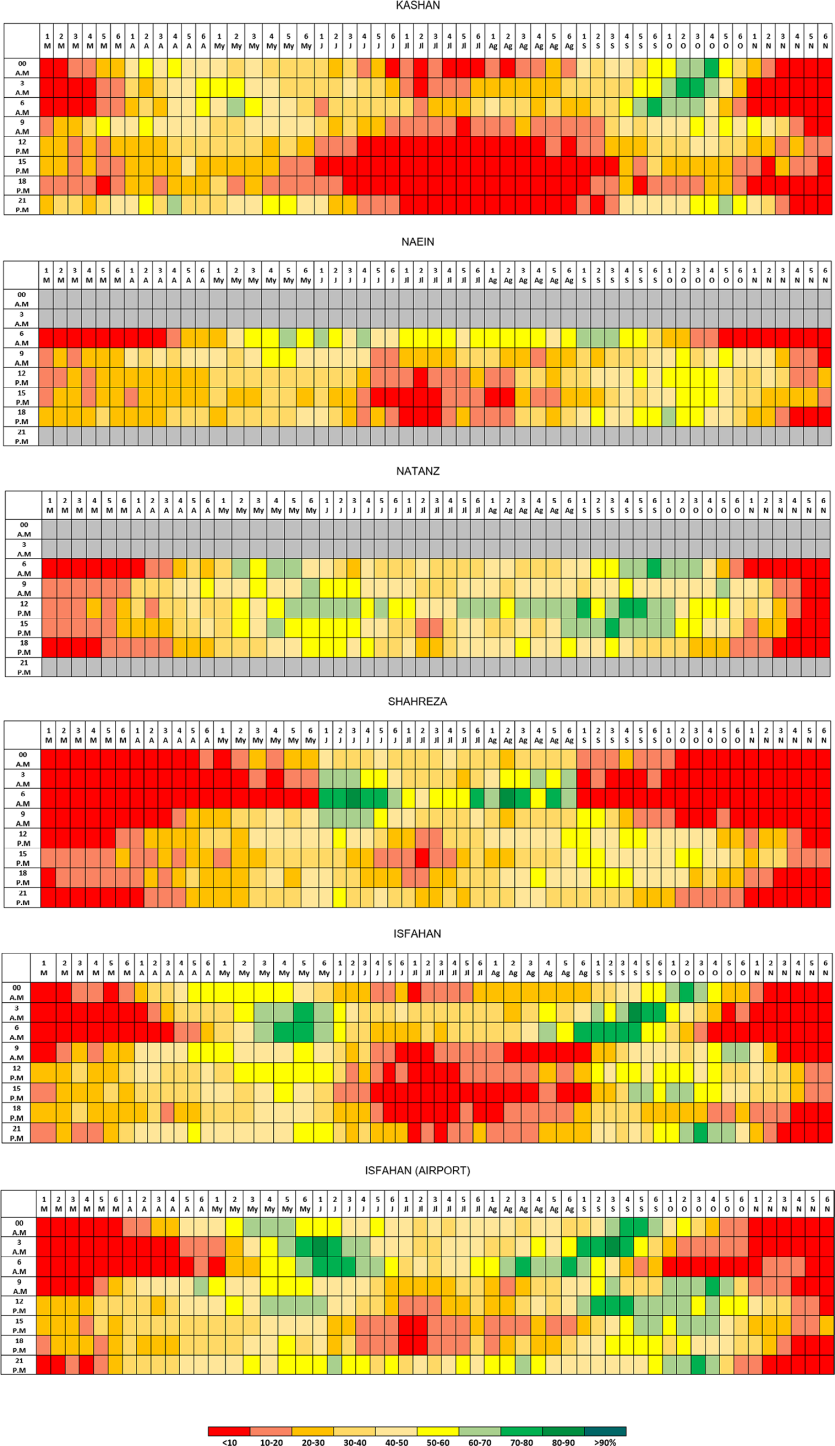
Percentage of favourable weather types for NBT in the province of Isfahan (hourly resolution)

The analysis using hourly resolution shows that the favourable conditions for nature-based tourism present significant intraday variations, although it is also the case that local and regional geographical diversity introduce nuances into the timetables for tourism climate potential that have been obtained at the synoptic observatories under study (see Fig. 2). These variations must be taken into account in the scheduling of leisure and recreational activities throughout the day and also in the organisation of annual activity calendars.

During the summer, the observatory at Kashan (the one at the lowest altitude) followed by the observatory at Isfahan (an urban observatory) are the two that show the least suitability for nature-based tourism. The favourable conditions are below 10% in Kashan and below 20% in Isfahan in practically every time band in the period spanning from early June to late August (and the same conditions even stretch into the first half of September). The only exceptions are the time band from 0600 to 0900 local time in Kashan and the time band between 0300 and 0900 in Isfahan, which all present frequencies of favourable types that fall largely between 20% and 40%. In the two locations, therefore, nature-based tourism can be pursued in the form of early morning excursions, whereas it would be advisable across the remaining time bands to pursue activities in places regarded as “climate shelters” or in venues located indoors. In both cases, the weather conditions in summertime limit the pursuit of weather-based tourism to very specific narrow windows of time. In any event, we must take into account the influence of urbanisation on the modification of the regional climate in the case of the Isfahan observatory (which is, as noted earlier, an urban observatory), so the interpretation of the results ought to be supplemented with the results gathered from the observatory at Isfahan Airport, which is more representative of the regional climate.

More specifically, the summertime conditions recorded in the observatory at Isfahan Airport are highly favourable for nature-based tourism (20–40% in the middle of summer and 40–80% at either ends of summer), with the exception of the time band of 1200–1800 local time in the period from 20 June to 25 July, when the values fall below 20% because of the high recorded temperatures and the occurrence of wind speeds greater than the values regarded as acceptable by Iranians. During the summer period, the behaviour at Naein and Shahreza is very similar to the behaviour at Isfahan Airport. The observatory at Natanz presents the greatest suitability for nature-based tourism, although the lack of hourly information at 2100, 0000, 0300 and 0600 local time limits the analysis of results.

In Shahreza, the tourism climate potential in springtime is low (below 20%) in practically every time band in the months of March and April and also in the time band between 0000 and 0900 in May (so that activities would instead be feasible later in the mornings and afternoons). At Natanz and Isfahan Airport, the month of March has a low suitability (the frequencies of favourable weather types are lower than 20%), while April and May show a moderate suitability (largely 30–60%) throughout the entire day. In the remaining observatories, the middle time band of the day in the month of March presents favourable frequencies for nature-based tourism between 20% and 40%, whereas these values and even higher values are recorded throughout the entire day in April and May.

In Shahreza, the tourism climate potential in autumn is very low (below 10%) in practically every time band in November, and it is low (10–20%) in the time band from 2100 to 1200 in the months of October and September. The autumnal conditions in the remaining observatories present similar behaviour in November, but less markedly so. Broadly speaking, however, the months of October and November at the remaining observatories are highly favourable for nature-based tourism throughout the entire day (with frequencies between 40% and 70% and even between 70% and 90%), making October and November (together with April and May) the most suitable months for this sort of tourism.

## Discussion and conclusions

The present study is the first to evaluate the tourism climate suitability of arid regions applying hourly resolution with the weather types method. The weather types method is a good tool for evaluating the tourism climate potential of a destination, because it incorporates a combination of different atmospheric variables that make up the actual weather at any given time. Weather types, which are identified as real atmospheric situations, are significant not only for tourists but also for tourism planners and operators. As a result, they are distinct from the tourism climate indexes that are traditionally used in studies of tourism climatology. While such indexes furnish overall suitability values that are easy to interpret, they rarely identify real atmospheric situations (Besancenot 1991; De Freitas 2003; Matzarakis 2014).

The weather types method applied in the present study makes it possible to produce a catalogue of weather types favourable for tourism, taking into account not only the climate reality of tourism regions but also any bioclimatic criteria and preferences of specific segments of tourists. In addition, the method considers the three facets of climate (physical, thermal and aesthetic) and it is possible to apply hourly resolution since the weather experienced by tourists coincides with the actual state of the atmosphere that occurs at any given time and place, as reflected in the information provided by synoptic observatories in the region in question. If all of these aspects are taken into account, it is possible to say that the application of the proposed weather types method is not affected by the various limitations that beset other climate indexes used in tourism research (Scott et al. 2016; Rutty et al. 2020): the absence of empirical verification of the scales for rating and weighting the variables; the failure to assess the dominance of physical variables over aesthetic and/or thermal variables; the failure to consider a variety of existing demand segments and their specific weather-related requirements; and the limited temporal resolution of the climate variables used in the indexes. The value of the frequency (in %) of favourable weather types is a good indicator of tourism suitability.

By enabling work at a more detailed temporal resolution, the weather types method is good for evaluating the tourism suitability of arid regions: the significant range of daily temperatures in arid settings and the associated wind patterns support the advisability of analysis at an hourly resolution to complement the daily resolution. The present study has shown clearly that applying daily resolution and considering average values or values referring to the middle hours of the day may well be masking favourable conditions that fall outside the midpoint of the standard period of recreational activity. This fact proves to be of great interest since many studies have shown that the extent of daily activity that can be pursued outdoors by tourists at a destination takes into account neither the value of the average daily temperature nor the value of the daily high temperature, but rather the distribution of temperatures throughout the day; and the same thing occurs with other variables such as the wind, precipitation and relative humidity (Dundas and von Haefen 2020; Fan et al. 2023). Applying hourly resolution enables the identification of time bands favourable for the pursuit of tourism activities within periods that have been found to be unfavourable when applying daily resolution (Nourmohammadi Najafabadi and Gómez-Martín 2023). Similarly, using hourly resolution has also identified unfavourable time bands that have been identified as favourable in evaluations using daily resolution (Nourmohammadi Najafabadi and Gómez-Martín 2023). Applying hourly resolution in the analysis has shown that time bands in the early morning, in the late afternoon and at dusk (and even, in some cases, at night) are suitable periods for outdoor activities in many of the analysed destinations at the height of the summer season. Also, the opposite occurs in the early spring (March) and late autumn (November), since it is the middle hours of the day that offer the most suitable conditions. Lastly, the months of April-May and October-September offer the greatest possibilities for outdoor activity throughout the entire day. Consideration of this information would enable destinations with extreme arid climates of Isfahan province to be more competitive in the market by offering distinct leisure and recreational activities in accordance with the environmental conditions at any given time of the day and year.

These findings could have a positive influence on the quality of organisational strategies and decision-making in destinations in arid climates by helping to determine the time bands when it is possible to engage in leisure and recreational activities related to nature-based tourism outdoors (with different intensities of physical activity) and the time bands when it is advisable to seek shelter from the climate and pursue activities indoors. Applying hourly resolution provides detailed information that can enable not only tourists but also tourism managers to establish strategies of intraday adaptation for the pursuit of activities even in regions with extreme climates. After all, adaptation to extreme conditions makes it possible for tourists to avoid physical and mental stress that can have an influence on their health, enjoyment and satisfaction.

To the best of our knowledge, the present study is the first to apply hourly resolution with the weather types method in arid climates. While the results clearly show the informational advantages of applying the approach, it is also important to note a few limitations with respect to the present and the future. First, there are limitations related to the failure to include some atmospheric phenomena or variables of major significance in the context of arid environments (e.g. haze and visibility, to name but two examples). Addressing this shortfall could offer a future line of research. Second, this fine resolution of work cannot always be carried out due to the characteristics and state of the conventional and automatic observation stations in certain places in the world. This problem is always implicit in the use of climatic and meteorological data, regardless of the type of source used. Therefore, the improvement of databases for the generation of products and provision of services must be a priority in the policies of the organizations in charge of managing climate data. Third, while applying hourly resolution has clear advantages in evaluating the tourism potential of present climates, it may prove hard to carry out in the evaluation of future climates (at least for now).

Against the backdrop of worsen weather conditions in a wide range of tourism destinations worldwide as a consequence of climate change, it would seem advisable to supplement traditional analyses of tourism climate potential that use daily resolution with analyses that apply hourly resolution. Such an approach would enable planners and operators to introduce adaptive measures for the survival of tourism activity and the resilience of destinations affected by the deterioration in weather conditions.
